# CD11b^+^Ly6C^++^Ly6G^-^ cells show distinct function in mice with chronic inflammation or tumor burden

**DOI:** 10.1186/1471-2172-13-69

**Published:** 2012-12-12

**Authors:** Eva Källberg, Martin Stenström, David Liberg, Fredrik Ivars, Tomas Leanderson

**Affiliations:** 1 Immunology Group, Lund University and Active Biotech AB*, BMC D14, SE-22184, Lund, Sweden

**Keywords:** Tumor, Inflammation, Myeloid cells, T cells, Suppression

## Abstract

**Background:**

S100A9 has been shown to be important for the function of so called Myeloid Derived Suppressor Cells (MDSC). Cells with a similar phenotype are also involved in pro-inflammatory processes, and we therefore wanted to investigate the gene expression and function of these cells in animals that were either subjected to chronic inflammation, or inoculated with tumors.

**Methods:**

CD11b^+^Ly6C^++^ and Ly6G^+^ cells were isolated from spleen, tumor tissue or inflammatory granulomas. S100A9, Arginase 1 and iNOS gene expression in the various CD11b^+^ cell populations was analyzed using Q-PCR. The suppressive activity of the CD11b^+^ cell populations from different donors was studied in co-culture experiments.

**Results:**

S100A9 was shown to be expressed mainly in splenic CD11b^+^Ly6C^+^G^+^ cells both at the RNA and protein level. Arginase I and iNOS expression could be detected in both CD11b^+^Ly6C^+^Ly6G^+^ and CD11b^+^Ly6C^+^G^-^/C^++^G^-^ derived from tumors or a site of chronic inflammation, but was very low in the same cell populations isolated from the spleen. CD11b^+^ cells isolated from mice with peritoneal chronic inflammation were able to stimulate T lymphocytes, while CD11b^+^ cells from mice with peritoneal tumors suppressed T cell growth.

**Conclusion:**

An identical CD11b^+^Ly6C^++^G^-^ cell population appears to have the ability to adopt immune stimulatory or immune suppressive functions dependent on the presence of a local inflammatory or tumor microenvironment. Thus, there is a functional plasticity in the CD11b^+^Ly6C^++^G^-^ cell population that cannot be distinguished with the current molecular markers.

## Background

S100A9 is a Ca^++^ binding protein that shows elevated serum levels in patients with inflammatory diseases [1], but also in some cancer patients [2]. In serum, S100A9 is mostly present as a heterodimer together with another S100 protein, S100A8 [3]. S100A9 and S100A8 are expressed in high amounts in neutrophils but also in immature monocytes [4].

Various biological functions have been ascribed to S100A9. Hence, S100A9 has been shown to bind to Toll Like Receptor 4 (TLR4) and to the Receptor of Advanced Glycation End products (RAGE) [5]. Both of these receptors have been shown to be involved in inflammatory responses, but also to promote tumor growth [6,7]. S100A9 has also been shown to be involved in the process of tumor metastasis [8]. In a recent publication, we investigated the impact of S100A9- and TLR-4 deficiency on tumor growth in the TRAMP prostate cancer model [9]. Our results demonstrated a significant delay of tumor growth in these animals. We obtained similar data on tumor growth when S100A9-deficient (S100A9^−/−^) and TLR4^−/−^ mice were transplanted with EL4 lymphoma cells. As EL4 tumor growth was not reduced in RAGE^−/−^ mice, we concluded that the interaction of S100A9 with TLR4 promotes tumor growth. This conclusion was supported by the finding that ABR-215050, a small molecule inhibitor of the S100A9/TLR4 interaction, significantly inhibited EL4 tumor growth [9].

Interestingly, S100A9 has also been shown to be important for the function of so called Myeloid Derived Suppressor Cells (MDSC) [10]. These cells consist of a heterogeneous population of myeloid cells that is usually described as being CD11b^+^ and GR1^+^ cells [11]. The GR1 marker is a composite epitope between the Ly6C and Ly6G antigens, and MDSC can be further subdivided into Ly6C^++^ monocytic and Ly6G^+^ granulocytic MDSCs using these two antigens [10]. The MDSC population promotes tumor growth by several different mechanisms, amongst these by expressing the arginine metabolizing enzyme Arginase I (Arg I) and inducible nitric oxide synthase (iNOS) [11-13]. Expression of these enzymes provides strong immunosuppression by inhibiting T cell activity and thus the adaptive immune response towards the tumor. CD11b^+^Ly6C^++^ and Ly6G^+^ cells are also involved in pro-inflammatory processes and we therefore wanted to investigate the gene expression and function of these cells in animals that were either subjected to chronic inflammation, or inoculated with tumors.

We show in this communication that although isolated CD11b^+^ subpopulations cannot be distinguished by their expression of S100A9, Arg I or iNOS in the two settings, they are stimulatory to T cells when isolated from spleens of mice with chronic inflammation while being immune suppressive when isolated from mice with tumors. Thus, there is a functional plasticity in the CD11b^+^Gr1^+^ population that cannot be distinguished with the current molecular markers.

## Methods

### EL-4 lymphoma model

All animal experiments have been approved by a local ethics committee (“Malmö/Lunds Djurförsöksetiska nämnd”; DNR M 275–08 and M 60–10). C57BL/6 mice, (Taconic M&B, Ry, Denmark) mice were kept in an SPF animal facility at BMC, Lund. Twelve weeks old animals were injected subcutaneously with 50,000 EL4 lymphoma cells in 100 μl PBS. As control, 100 μl PBS alone was injected. After 14 days the animals were scored for tumor growth by palpation. Spleens and tumors were dissected, the cell suspension was thereafter passed through a 70 μm cell strainer and cells washed in Hank’s BSS (Invitrogen Life Technologies, Paisley, UK). When indicated 20,000 EL-4 cells were injected IP and after 10 days peritoneal cavity lavage were used as source for CD11b^+^ cells.

### TMPD model

All TMPD animal experiments were conducted in the animal facility at Active Biotech AB. Eight to nine weeks old female BALB/c mice (Taconic, Denmark) were given a single intraperitoneal injection (0.5 ml) of 2,6,10,14-Tetramethyl-Pentadecane (TMPD, pristane; Sigma-Aldrich, US). Two weeks after TMPD administration the spleen, peritoneal cells and granulomas were harvested. The spleen was mashed through a 70 μm cell strainer. The peritoneal cavity was lavaged with 5 ml PBS and cells were collected by centrifugation. The granulomas were picked from the peritoneal lining and mashed through a 70 μm cell strainer. All cell samples were re-suspended in RPMI 1640 supplemented with 10% FCS.

### Cell culture conditions

CD11b^+^ cells were enriched from C57Bl/6 or BALB/c spleens with magnetic cell sorting (MACS; Miltenyi Biotec, Bergisch Gladbach, Germany), performed according to the manufacturer’s protocol, using anti-CD11b magnetic beads (Miltenyi). The cells were separated on mini MACS columns (Miltenyi) and yielded approximately 90% pure cells. Splenic CD4 and CD11c positive cells were enriched in the same way using anti-CD4 or anti-CD11c antibody-conjugated beads.

Suppressor cell activity was assessed by co-culturing various numbers of CD11b^*+*^ cells with 5 × 10^4^ CD4^+^ cells and 3 × 10^3^ CD11c^+^ cells in 200 μl cultures in round-bottom 96-well plates (Costar, Cambridge, MA). T cells were polyclonally stimulated by the addition of 1 μg/ml anti-CD3 antibodies (145.2C11) and 1 μg/ml anti-CD28 to the cultures. Cells were cultured in RPMI medium (Gibco) supplemented with 50 μM 2-ME, antibiotics, 10% FCS, 1 mM sodium pyruvate and 10 mM Hepes buffer (all supplements from Gibco) at 37°C, 5% CO_2_. Thymidine incorporation was measured on day 3 of culture after a 4-h pulse with 1 μCi [^3^H] thymidine (Amersham, Life Science).

### Q-PCR

Splenic CD11b^+^ cells were purified using anti-CD11b magnetic beads and LS-columns (Miltenyi Biotech, Bergisch Gladbach, Germany), as described above. Total RNA was extracted from CD11b^+^ cell preparations by use of the Purelink RNA mini Kit (Invitrogen). RNA was reverse transcribed to cDNA by use of the SuperScript III Platinum synthesis system (Invitrogen). Real-time PCR (RT-PCR) was performed for the detection of S100A9, Arginase and iNOS RNA and quantified using a SYBR GreenER kit (Invitrogen) in a MYIQ (Bio-Rad) PCR machine. The threshold cycle number was determined and relative expression level of each mRNA was determined using the formula 2^(Rt– Et)^, where Rt and Et are the threshold cycles for the reference gene (β-actin) and the target gene, respectively.

### Flow cytometry

Flow cytometric analysis was performed on spleen cell suspensions, as indicated. Primary antibodies used were: anti-mouse CD11b-APC (eBioscience), Ly6G-FITC (BD Pharmingen) and Ly6C-biotin (BD Pharmingen). Biotinylated antibodies were detected with streptavidin-QD605 (Invitrogen). Data were acquired using a FACS LSR II flow cytometer (BD Biosciences) and analyzed using FlowJo software (Tree Star).

### Immunohistochemistry

Tissues analyzed with immunohistology were embedded in OCT compound (Tissue-Tek®), and snap-frozen in liquid nitrogen. Cryosections (5–6 μm) were prepared on microscope slides, air dried and frozen at −20°C until staining procedures. Paraformaldehyde fixed sections were incubated with blocking 1% BSA 10% serum and FcRII/III blocker solution followed by Avidin/Biotin Blocking kit (Vector Laboratories, Inc. Burlingame, CA, USA). Thereafter the sections were incubated for 30 min at room temperature with primary antibodies: Rabbit-anti-murine S100A9, or the appropriate isotype controls (BD Pharmingen), followed by Donkey- anti- rabbit-Alexa488 (Molecular Probes) and anti-mouse CD11b-APC conjugate (eBioscience San Diego CA, USA), Ly6G-PE (BD Pharmingen), Ly6C-biotin (BD Pharmingen) followed by Streptavedin labeled with Alexa-647 (BD Pharmingen). The slides were mounted using ProLong Gold mounting media (Invitrogen, Oregon, USA) and inspected in a Zeiss microscope and analyzed with Volocity software.

### Western blot

Spleen cells were stained as described above and Ly6C^+^G^+^, Ly6C^+^G^-^ and Ly6C^++^G^-^ subpopulations were sorted using a FACSAria flow cytometer (BD Biosciences). For Western blot, 10 μg of proteins was loaded onto 12% polyacrylamide gels (C.B.S. Scientific, San Diego, CA, USA). Proteins were subsequently transferred to PVDF membrane (Roche), which was saturated with 1% dry milk in PBS. Thereafter, the membranes were incubated with Rat anti-Mouse S100A9 and Rat anti-Actin (RnD Systems) as primary antibody and Rabbit anti-Rat –HRP (SouthernBiotec Birmingham, Alabama, USA) as secondary antibodies and filters developed using ECL kit (GE Healthcare, UK).

## Results

### S100A9 expression in splenic CD11b^+^ cells

**Figure 1:**
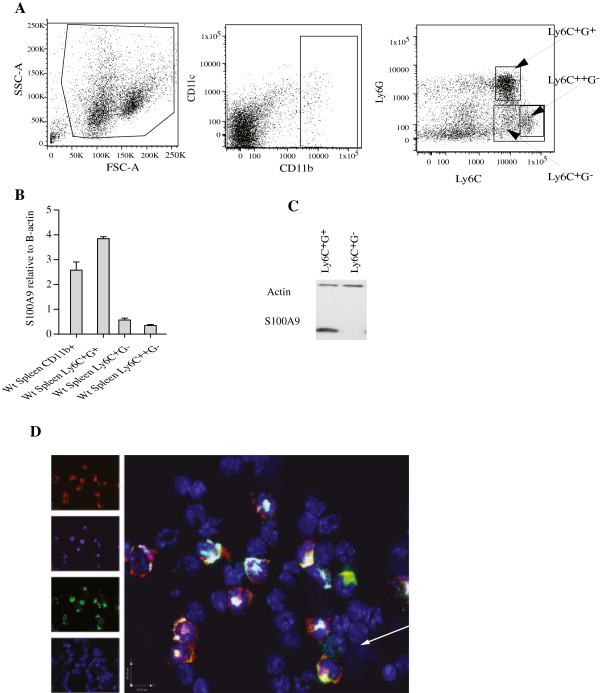
**Expression of S100A9 in the splenic CD11b**^**+**^**cells.A.** Dot-plots from FACS analysis to illustrate the gating to obtain the different CD11b^+^ subpopulations. **B.** RT-PCR analysis of S100A9 RNA expression in FACS sorted cells from spleen, as indicated. **C.** Western blot analysis of S100A9 expression on FACS sorted cell populations from spleen. **D.** Cytospin of splenic CD11b sorted cells were stained with anti-Ly6G in red, anti-Ly6C in purple, anti-S100A9 in green and Hoecst in blue.

S100A9 has been shown to be involved in the function and accumulation of MDSC [10] and we first wanted to analyze the expression of S100A9 in various subsets of CD11b^+^ cells from the spleen of normal C57BL/6 mice in order to define whether there was a selective expression in a defined cell subset. To this end, we sorted CD11b^+^ cells using FACS with regard to the expression of the Ly6G and Ly6C markers into three populations; Ly6C^+^G^+^, Ly6C^+^G^-^ and Ly6C^++^G^-^, as shown in Figure 1A. RNA was prepared from the isolated cell populations and S100A9 expression was measured using Q-PCR. As shown in Figure 1B, the Ly6C^+^G^+^ population appeared to be the main S100A9 expressing subpopulation within the CD11b^+^ compartment. This was confirmed at the protein level using Western blotting (Figure 1C). Furthermore, when CD11b^+^ cells were stained and analyzed using immunohistochemistry, the majority of the S100A9^+^ cells also stained brightly for Ly6G while Ly6C^+^ cells only stained weakly, if at all, with anti-S100A9 (Figure 1D). Thus, S100A9 is differentially expressed within the CD11b^+^Gr1^+^ cell compartment where the Ly6C^+^G^+^ cells is the main S100A9 expressing cell population.

### Effect of tumor on CD11b^+^ cell populations and S100A9

**Figure 2:**
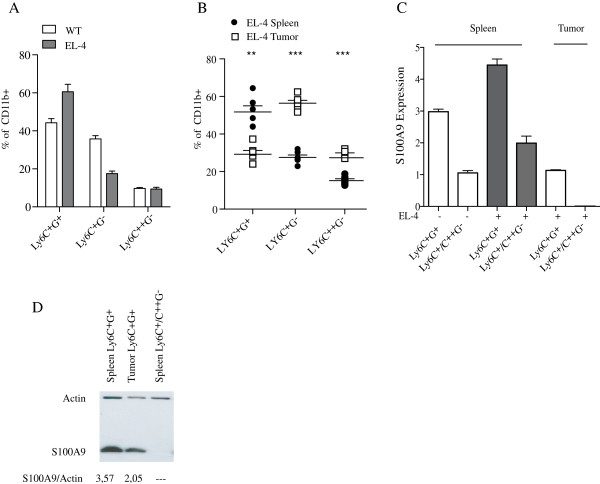
**Tumor microenvironment induces a shift in CD11b**^**+**^**cell populations.A**. Proportion of splenic CD11b^+^ cell population in C57BL/6 control animals and animals inoculated 14 days earlier with EL-4 lymphoma cells. **B**. Proportion of different CD11b^+^ cell populations in spleen and tumor from EL-4 inoculated C57BL/6 mice. **C**. RT-PCR analysis of S100A9 RNA expression in sorted cells from spleen and EL-4 tumors, as indicated. The threshold cycle number was determined and relative expression compared to the reference gene (β-actin) and the target gene, respectively. **D**. Western blot analysis of S100A9 expression on sorted cell populations from spleen and EL-4 tumors, as indicated. The Gray Value (Median) from Adobe Photoshop were used to determined the level of S100A9 and the reference gene (β-actin) expression. Statistical analysis was performed using Student’s *t*-test (*p < 0.05; **p < 0.01;***p < 0.001).

We next analyzed the effect of a tumor challenge on the CD11b^+^ cell populations and their respective S100A9 expression. C57BL/6 mice were inoculated with EL4 lymphoma cells subcutaneously and 14 days after the inoculum the splenic CD11b^+^ cells were analyzed by FACS, as above. As shown in Figure 2A, the percentage of CD11b^+^Ly6C^+^G^+^ increased while CD11b^+^Ly6C^+^G^-^ cells in the spleen decreased in tumor bearing animals. The composition of the CD11b^+^ cells in the spleen and in tumor tissue differed. Hence, the CD11b^+^Ly6C^+^G^+^ population was reduced in the tumor while the CD11b^+^Ly6C^+^G^-^ population was overrepresented (Figure 2B). The CD11b^+^Ly6C^++^G^-^ population was also slightly elevated in the EL-4 tumors.

We next wanted to investigate the effect of tumor inoculation on S100A9 expression (Figure 2C). In order to get enough cells from tumor tissue to be able to extract sufficient amount of RNA for analysis, we pooled the CD11b^+^Ly6C^+^G^-^ and CD11b^+^Ly6C^++^G^-^ populations. There was a modest increase of S100A9 mRNA in both splenic populations (CD11b^+^Ly6C^+^G^+^ and CD11b^+^Ly6C^+^G^-^/C^++^G^-^) in EL-4 tumor bearing animals. However, when cells with the same surface phenotype were isolated from the tumor tissue, the S100A9 RNA expression was lower compared to the corresponding splenic cell populations. Using Western blot (Figure 2D) the down-regulation of S100A9 could also be seen in tumor-derived CD11b^+^Ly6C^+^G^+^ cells at the protein level, although not as marked as at the RNA level. We conclude from these experiments that the distribution of CD11b^+^ cell subpopulations, as well as their S100A9 expression, appears to be dynamic and change both depending on tumor challenge and on tissue location.

### Differential S100A9 expression during chronic inflammation

**Figure 3:**
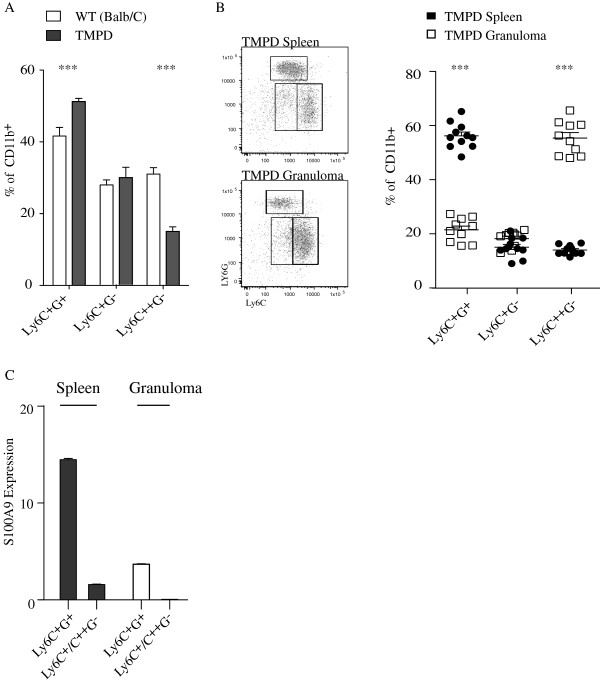
**Chronic inflammatory microenvironment induces a shift of CD11b**^**+**^**cell populations.A**. Proportion of splenic CD11b^+^ cell population in BALB/c control animals and animals inoculated 14 days earlier with pristane i.p. **B**. Proportion of different CD11b^+^ cell populations in spleen and granuloma inoculated 14 days earlier with pristane i.p. **C**. RT-PCR analysis of S100A9 RNA expression in sorted cells from spleen and granulomas from animals inoculated 14 days earlier with pristane i.p., as indicated. The threshold cycle number was determined and relative expression compared to the reference gene (β-actin) and the target gene, respectively. Statistical analysis was performed using Student’s *t*-test (*p < 0.05; **p < 0.01;***p < 0.001).

Having investigated the effect of a tumor challenge on the CD11b^+^Gr1^+^ cell distribution and S100A9 expression we decided to compare this to what was observed in chronic inflammation. To this end we used a mouse model where injection of TMPD (pristane) into the peritoneum induces granuloma formation, ultimately resulting in a SLE-like disease [14]. The mice were sacrificed 14 days after TMPD injection and the CD11b^+^ cells from the spleens and granulomas were analyzed using FACS and Q-PCR. We first investigated the effect on the CD11b^+^ cell populations in the spleen in BALB/c control animals and TMPD injected animals. Here, the CD11b^+^Ly6C^+^G^+^ cell population increased, while the CD11b^+^Ly6C^++^G^-^ population decreased upon TMPD injection (Figure 3A). Furthermore, the CD11b^+^ population was analyzed also in granulomas with respect to the expression of the Ly6C and Ly6G markers. The Ly6C^+^G^+^ population was reduced in granulomas compared to spleen, the Ly6C^+^G^-^ population similar, while the Ly6C^++^G^-^ population was elevated in granulomas (Figure 3B). Thus, with regard to these markers, different subpopulations of CD11b^+^ cells appear to accumulate at the site of TMPD-induced chronic inflammation as compared to tumor tissue.

We also analyzed the S100A9 expression in the different CD11b^+^ subpopulations using Q-PCR (Figure 3C). In the spleen, the most pronounced S100A9 expression was in the Ly6C^+^G^+^ population, similarly as to what was observed in tumor bearing animals. Also, the S100A9 expression in this cell population was down-regulated in the granulomas, as observed in tumor tissue.

### Functional properties of CD11b^+^ cell populations from tumors or inflamed tissue

**Figure 4:**
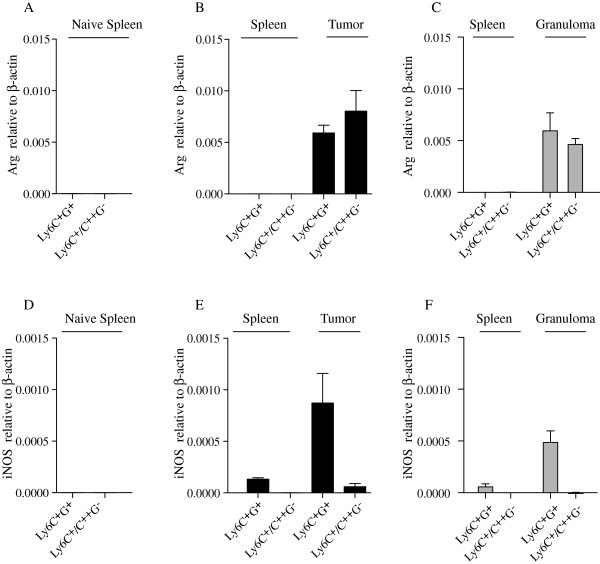
**Arginase and iNOS expression are very similar in CD11b**^**+**^**cell subpopulations from tumors or chronic inflammatory microenvironments.** Indicated CD11b^+^ cell populations were isolated by cell sorting from spleen of control naïve mice (**A**, **D**), or from spleen and tumor tissue of mice carrying EL4 tumor (**B**,**E**) or from spleen and granuloma tissue of mice carrying peritoneal granulomas (**C**,**F**). RNA was isolated from the sorted cells and qRT-PCR was performed to determine expression of Arginase I (Arg) or iNOS relative to β-actin expression, as indicated. qRT-PCR was performed in triplicate and data displayed as mean expression +/− SD. The same mRNA preparations were analyzed in **A**-**C** and **D**-**F.**

We next wanted to gain some insight into the functional properties of the CD11b^+^ subpopulations under different conditions. Firstly, we analyzed arginase-I (Arg) and iNOS RNA expression from the CD11b^+^ subpopulations using RT-PCR. As shown in Figure 4A-C, very low Arg expression was detected in both CD11b^+^ Ly6C^+^G^+^ and Ly6C^+^G^-^/C^++^G^-^ cells isolated from the spleen of either control animals or animals with EL4 tumors or granulomas. However, significant Arg expression was readily detected in the same cell populations isolated from either tumor tissue or granulomas. With regard to iNOS expression, a similar picture emerged (Figure 4D-F); both Ly6C^+^G^+^ and Ly6C^+^G^-^/C^++^G^-^ cells from tumors and granulomas expressed iNOS RNA. The highest expression was seen in the Ly6C^+^G^+^ population in both cases. Also, no iNOS expression was detected in spleen cells from control animals while a low expression could be detected in the CD11b^+^Ly6C^+^G^+^ population from spleens of animals carrying either tumors or granulomas. We conclude from these experiments that the expression of the immune regulatory molecules Arg and iNOS appears to be up-regulated in a similar way in peripheral tissue, both in animals challenged with chronic inflammation or tumors.

**Figure 5:**
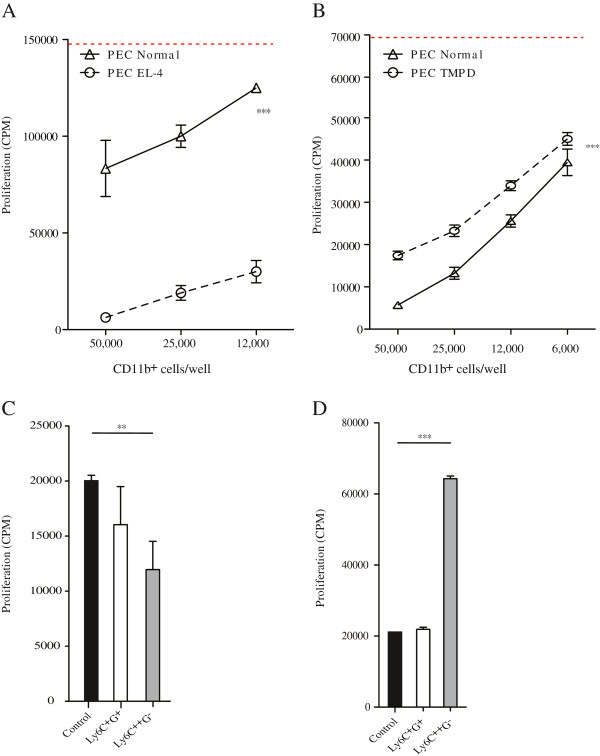
**Effect of CD11b**^**+**^**cell populations on T cell proliferation *in vitro*.A**. Indicated numbers of CD11b^+^ cells sorted from the peritoneal exudate cells (PEC) of normal control or EL-4 carrying C57BL/6 mice were co-cultured for 3 days with splenic CD4^+^ T cells and CD11c^+^ cells and proliferation induced with anti-CD3 and anti-CD28 antibodies. T cell proliferation was determined as described in Materials and Methods. **B**. CD11b^+^ cells sorted from peritoneal exudate cells (PEC) of normal control or granuloma-carrying BALB/c mice were co-cultured with splenic CD4^+^ T cells and CD11c^+^ cells isolated form BALB/c mice and proliferation determined as in **A**. Dashed red lines in **A** and **B** indicate unsuppressed T cell proliferation (in the absence of added CD11b^+^ cells). **C**. CD11b^+^ cell subpopulations (5x10^4^/culture), as indicated, sorted from spleens of EL-4 tumor inoculated C57BL/6 mice were co-cultured with splenic CD4^+^ T cells and CD11c^+^ cells as in **A**. **D**. CD11b^+^ cell subpopulations, as indicated, sorted from spleens of TMPD granuloma-carrying BALB/c mice were co-cultured with CD4^+^ T cells and CD11c^+^ cells isolated BALB/c mice, as described in **C**. Proliferation in control cultures without added CD11b^+^ cells were analysed in parallel. The data shows mean +/− SD of triplicate cultures. Statistical analysis was performed using two-way ANOVA (** p < 0.01; *** p < 0.001) in panels **A** and **B**, and Student *t*-test (*p < 0.05; **p < 0.01; ***p < 0.001) in panels **C** and **D.**

To further address the functionality of the CD11b^+^ cells in various conditions we performed co-culture experiments with splenic CD4^+^ T cells stimulated with anti-CD3 and anti-CD28 antibodies in the presence of CD11c^+^ dendritic cells, as previously described [10]. The indicated CD11b^+^ subpopulations were added to the cultures and cell proliferation was measured after three days by ^3^H-thymidine uptake. We first isolated CD11b^+^ cells from peritoneal lavage (PEC) from animals with induced granulomas, or from animals inoculated with EL-4 tumors intra-peritoneally, and used them in T cell proliferation assays. As shown in Figure 5A, peritoneal CD11b^+^ cells from EL-4 tumor inoculated animals were highly immune suppressive compared to CD11b^+^ cells isolated from control animals that suppressed to a lesser extent. Peritoneal CD11b^+^ cells from animals with granulomas, on the other hand displayed even less suppression than control cells from normal mice (Figure 5B). Hence, a functional distinction between CD11b^+^ cells isolated from animals with EL-4 tumors or TMPD-induced chronic inflammation could be detected with regard to their ability to influence T cell proliferation.

We then wanted to study the ability of splenic CD11b^+^ cellular subsets to suppress T cell proliferation. As shown in Figure 5C, using FACS-separated splenic CD11b^+^ cells from EL-4 tumor inoculated animals, some immune suppressive activity was observed when adding CD11b^+^Ly6C^+^G^+^ cells, as compared to the control cultures where no CD11b^+^ cells were added. The suppressive effect was even more pronounced when CD11b^+^Ly6C^++^G^-^ cells were added to the cultures. In contrast, when CD11b^+^Ly6C^++^G^-^ cells were FACS-sorted from the spleen of animals with chronic inflammation, these cells were immune stimulatory (Figure 5D), while CD11b^+^Ly6C^+^G^+^ cells did not influence the *in vitro* T cell proliferation in this assay.

## Discussion

In this paper we investigated the function and gene expression pattern of various CD11b^+^ cellular subsets. We found that although expressing the same surface phenotype these cell populations were functionally different, with respect to their capacity to influence T cell proliferation, when they were isolated from tumor bearing animals compared to animals with chronic inflammation. However, the gene expression profile of both the Ly6C^+^G^+^ and Ly6C^+^G^-^/Ly6C^++^G^-^ CD11b^+^ cells was surprisingly similar between the two disease models. It has been shown that CD11b^+^Ly6C^++^G^-^ cells, which represent inflammatory monocytes, can be recruited both to tumor sites [15] and to sites of chronic inflammation [16,17]. Previously published data have indicated that monocytic MDSCs (CD11b^+^Ly6C^++^Ly6G^-^) suppress T cell proliferation through an iNOS dependent, Stat-1 dependent mechanism [18,19]. In the present study we saw no correlation between iNOS mRNA content and suppressive function of the splenic CD11b^+^Ly6C^++^Ly6G^-^ cells. We speculate that iNOS activity of cells derived from tumor-bearing mice may be boosted by T cell-produced IFNγ both *in vivo* and *in vitro*[20,21], which might explain this discrepancy.

The CD11b^+^Ly6C^++^Ly6G^-^ population had expanded extensively in the spleen of granuloma-carrying mice. In contrast, mainly the CD11b^+^Ly6C^+^G^+^ population, and to a lower extent the CD11b^+^Ly6C^++^Ly6G^-^ population, expanded in spleen of mice with experimental autoimmune encephalomyelitis (EAE) [22]. The myeloid cells expanding during this chronic inflammatory condition could be regarded as MDSCs [11], since they potently suppressed T cell proliferation [22]. The CD11b^+^ population expanded during the chronic inflammation in granuloma carrying mice analyzed in this study, could in analogy also be regarded as MDSCs. While MDSCs expand both during chronic and acute inflammatory conditions, it has been proposed that suppression of adaptive immunity may not be the only functional role of these cells (reviewed in [23]). For example, it has recently been shown that they promoted the induction of TH17 cells in EAE [24]. However, irrespective of whether the myeloid cells in granuloma carrying mice do represent *bona fide* MDSCs, or not, the observation that the monocyte-like CD11b^+^Ly6C^++^Ly6G^-^ cells isolated from these mice promote, rather than suppress, T cell proliferation further underscores the functional plasticity of this cell population. Nevertheless, as previously observed in several different tumor models [11-13], we also found that CD11b^+^ cells isolated from tumors are profoundly immunosuppressive. Given the design of our assay, we assume that MDSCs suppressed T cells in a contact-dependent manner.

During the preparation of this manuscript Yi et al. published that classical MDSCs aggrevated experimental autoimmune encephalomyelitis (EAE) in mice [24]. This finding is in agreement with the data presented herein; namely that defined cell populations that have been described as immune suppressive in one disease setting (cancer) can have a different function in another (autoimmunity). With regard to the clinical implications these findings could open up for new treatments strategies. For example, gemcitabine, originally developed as a cytotoxic cancer treatment, has been shown to selectively deplete MDSCs [25]. Our observation, and that of Yi et al. [24], might indicate that gemcitabine also could be a treatment option for patients with aggressive, autoimmune/inflammatory disease.

## Conclusion

We show here that when the main immune regulatory population (CD11b^+^Ly6C^++^G^-^) in spleen was investigated for its ability to influence T cell growth, a marked difference was observed as cells from animals with chronic inflammation were stimulatory, while those from tumor bearing animals were immunosuppressive. Hence, the same cell population appears to have the ability to adopt an immune stimulatory or immune suppressive function dependent on the presence of a local inflammatory or tumor microenvironment, despite their common origin and phenotypic similarities.

## Competing interests

The authors declare that they have no competing interests.

## Authors’ contribution

TL, FI and DL planned the study and EK and MS performed the experiments. All authors analyzed and discussed the data. TL and FI wrote the manuscript with input from all the other authors. All authors have read and approved the final version of the manuscript.
